# Integration of social work into specialist palliative home service

**DOI:** 10.1177/26323524241310457

**Published:** 2025-01-26

**Authors:** Janice Lee Wartchow, Stefan Bär, Bernd Alt-Epping, Christina Gerlach

**Affiliations:** Heidelberg University Hospital – Department of Palliative Medicine, INF 305, Heidelberg, Baden-Württemberg 69120, Germany; Max Weber Institute of Sociology, Ruprecht-Karls-University Heidelberg, Germany; Heidelberg University Hospital – Department of Palliative Medicine, Germany; Heidelberg University Hospital – Department of Palliative Medicine, Germany

**Keywords:** homecare, integrated care, interprofessional, palliative care, social work, specialist palliative care

## Abstract

**Background:**

The specialist palliative home service (SAPV) federal framework contract for adults, to be enacted in Germany until 2028, does not legally mandate the hiring of a third professional group beyond specialist nurses and physicians, although palliative care embraces the psychosocial dimension and an interprofessional approach.

**Objectives:**

This article aims to explore the role of medical staff in integrating social work (SW) into SAPV.

**Design:**

Qualitative case study.

**Methods:**

The study utilised theoretical and qualitative quota sampling to explore barriers to integrating SW into SAPV-teams, ensuring diverse perspectives. Sequential analysis was applied to uncover collective interpretations, generating and validating interpretive hypotheses directly from the data.

**Results:**

Four physicians and four nurses from the SAPV-team based at Heidelberg University Hospital participated. The medical staff’s attributions to SW significantly impact its integration into SAPV. Their perception of the SW profession determines the extent and manner of its integration into daily practices. Attributions on SW in SAPV as determined by nurses and physicians were social-medical knowledge, counselling, being a core competence in SAPV and similarities to the profession of psychologists. In the examined case, the integration was effective, and there was a desire for an increased presence of SW because there is still a lack of their working hours, and the medical staff wished for the social workers’ presence during home visits.

**Conclusion:**

This study highlights that SAPV requires SW to be effective, nonetheless, not being considered in the new federal contract to allocate resources. Possible barriers against the integration of SW within the real-world clinical practice of palliative care should be further investigated in future studies by involving social workers’ and healthcare managers’ experiences and strategies to understand why the employment of social workers in SAPV is progressing slowly and inform strategies to enhance their integration.

## Plain language summary

How social work is included in specialist home palliative care services

In Germany, the specialist palliative home care (SAPV) contract for adults, effective until 2028, does not require hiring professionals beyond nurses and doctors. This study aims to understand how medical staff help integrate social work (SW) into SAPV. Using qualitative case study methods, semi-structured interviews were conducted with doctors and nurses, and sequential analysis was employed to uncover collective perspectives. Four doctors and four nurses from the SAPV-team at Heidelberg University Hospital participated. The medical staff’s views significantly influence how social work is integrated into SAPV. They decide how much and in what way SW is included in daily practices. They view SW as important for social-medical knowledge, guidance, and as a core part of SAPV, comparable to the role of psychologists. In this case, integration was effective, and there was a strong desire for more SW involvement due to limited working hours and a need for social workers during home visits. The study shows that SW is necessary for effective SAPV, but it is not considered in the new federal contract for resource allocation. In the case under review, the integration of SW into the SAPV-team has been implemented both formally and with room for negotiation. The team expressed a need for greater SW involvement, particularly due to limitations in working hours and the importance of having social workers present during home visits. This study demonstrates that the effectiveness of SW within the SAPV context is contingent on resource allocation under the new federal contract, as well as on the perceptions and ascriptions of the core medical staff.

## Introduction

Specialist palliative home care (SAPV) is crucial to ensure that patients receive appropriate care until the end of their lives. Although the WHO for a long time has been advocating for the integration of palliative care into standard health care,^1^ and despite the fact of 57 million people worldwide who need palliative care, only 14% receive it,^2^ SAPV is still not recognised as a public health priority in many countries. This lack of recognition contributes to significant inequalities in care. This is also the case in Germany and there is much to suggest that there is a gap in care.^3^

At the end of 2022, a federal framework agreement was introduced in Germany to standardise the SAPV system. Until then, the SAPV system, which has been a mandatory service provided by health insurance companies since 2007 and has experienced significant growth, was regulated separately for each federal state via 16 different model contracts.

Because it is not mandatory, the framework agreement’s aim to ensure consistent quality of palliative care regardless of the patient’s location lacks the inclusion of psychosocial care by trained specialists. Instead, psychosocial care is still often provided by volunteers or inadequately trained healthcare professionals,^4^ although it is acknowledged that there has been an increasing need for social workers in palliative care over the last decade.^4,5^

According to Deutsche Vereinigung für Soziale Arbeit im Gesundheitswesen e.V. – (German Association for Social Work in Healthcare), over 56,000 social workers are employed in various areas of the healthcare sector across Germany. In 2022, more than 23,100 social work (SW) positions remained unfilled nationwide. While this shortage extends across the entire healthcare sector, the unfilled social worker positions do not even include palliative care. Thus, the presence of social workers is not mandated by the new federal regulation, this highlights a critical gap in the area of SW in specialist palliative home care.^6,7^ After all, social workers could take on three main roles in palliative care: Addressing the psychological needs of clients and their families, promoting advanced care planning and providing bereavement counselling for grieving loved ones. In carrying out these activities, social workers could pay particular attention to vulnerable groups with special palliative care needs.^8^

Against this background, the general question arises of how the implementation of SW in SAPV can succeed in the absence of external regulation? Because SW must be incorporated into existing structures, the specific question is, how do health care personnel’s attributions affect the incorporation of SW into SAPV?

**Figure 1.**
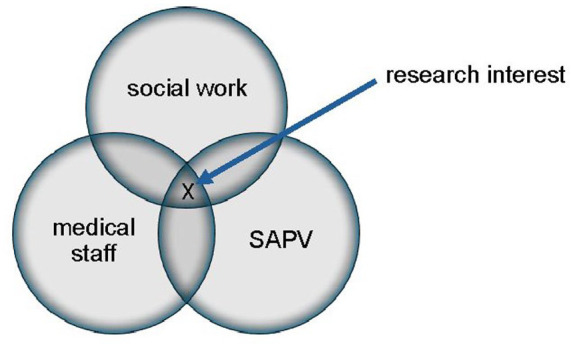
Research interest.

To answer these questions, we conducted a qualitative case study on the SAPV-team at the Heidelberg University Hospital, interviewing nurses and physicians to gain a better understanding of the facilitators and barriers to including social workers in SAPV-teams in Germany, as well as the unique skills they might bring to palliative medicine (see Figure 1).

The following paper presents the results of a qualitative case study based on interviews with doctors and nurses working in a SAPV-team at a German university hospital. We first describe the specific case under investigation and the empirical social research methods used. We then present the results of the analysis structured according to several dimensions. In the subsequent discussion section, we relate these to our research question and discuss the limitations of the study. In a brief concluding section, we summarise the main findings of the study.

This qualitative case study specifically addresses the following research question: How do medical personnel’s attributions affect the incorporation of SW into SAPV?

## Methods

The data presented in this article is based on one case study taken from J.W.’s master’s thesis research, which investigated the role of medical professionals in integrating SW into SAPV. The project was carried out at Heidelberg University in early 2024. During that time, she was working on the SAPV-team as well as studying sociology at Heidelberg University. The interviewees were aware that the study was part of her master’s thesis and that the purpose was to determine how they thought the implementation of SW had gone up until this point. Two researchers trained in participatory observation monitored the conduct of the interviews to prevent social desirability.

### Case description

The Department of Palliative Medicine, newly established in 2021 alongside the Chair of Palliative Medicine at Heidelberg University, delivers care through an interprofessional treatment model. It serves yearly approximately 320 patients in its dedicated palliative care unit at St. Vincentius Hospital, over 1100 patients through the interprofessional palliative care consultation service, more than 60 patients in the palliative care outpatient clinic established in 2022 at the National Center for Tumor Diseases, and more than 400 patients through the SAPV-team.

This SAPV-team has existed since long before the department itself. From 2013 to 2021, the team operated without a social worker. Since the department’s establishment, the SAPV-team now includes six doctors, nine nurses and one social worker. The social worker plays a key role in addressing the individual social needs of patients and their families, aiming to fully integrate SW into the team and support the interprofessional approach of palliative medicine.

In this case study, the integration of SW into a SAPV-team exemplifies the importance of interprofessional collaboration in palliative care. To address the concept of Total Pain, introduced by Cicely Saunders in 1996, which encompasses not only the physical but also the emotional, social and spiritual suffering of patients, a purely medical approach is insufficient.^9^ As palliative care moves forward, the integration of diverse professional roles, including SW, becomes essential to ensure holistic, patient-centred care. Collaboration among healthcare professionals is key to achieving synergy in care delivery, enhancing the quality, safety and efficiency of patient care.^10^ Interprofessional collaboration develops a caring culture in which team members’ fundamental needs are fulfilled, resulting in an atmosphere that brings healthcare professionals together in their common objective to offer the highest levels of care.^11^

## Sampling approach and analytical methods

The sampling process was initially guided by a form of sampling ensuring theoretical richness to address the primary objective of gaining insight into potential barriers to the integration of SW into a SAPV-team. It was deemed theoretically fruitful to engage with individuals who (a) had worked within the team prior to the introduction of a social worker and (b) those currently employed in the SAPV-team. This approach allowed for a nuanced understanding of the transition and integration process over time.

In the second phase, the sampling followed a qualitative quota sampling method, with the aim of covering all relevant dimensions of the phenomenon. The sample included representatives from all roles present in the case, deliberately varied by profession, hierarchy, age and gender to ensure potential contrast as shown in Table 1. However, the intent was not to achieve quantitative representativeness but rather qualitative depth, to capture the full spectrum of theoretically possible perspectives on SW that enabled a comparative analysis across different viewpoints within the team.^12–14^

**Table 1.** Socio-demographic characteristics.

Interview	Age	Profession	Years in the SAPV-team
I1	30–39	Physician	Less than three
I2	50–59	Physician	Less than three
I3	30–39	Nurse	Less than three
I4	50–59	Physician	Less than three
I5	50–59	Nurse	More than three
I6	30–39	Nurse	Less than three
I7	30–39	Physician	More than three
I8	50–59	Nurse	Less than three

SAPV, specialist palliative home service.

The method of sequential analysis was employed to reconstruct latent meanings and identify collective interpretations of SW within the SAPV context. The analysis also aimed to uncover the prevailing references to shared collective knowledge, drawing from the theoretical background of the sociology of knowledge and applied hermeneutics. Specifically, we sought to understand what underlying assumptions about SW existed, in what variations they manifested, and which perspectives were dominant. By applying this method, the goal was to develop interpretive hypotheses and validate them through cross-comparison.^15,16^ It is important to note that this approach was not focused on the empirical testing of pre-established hypotheses. Instead, it aimed at validating interpretive hypotheses generated directly from the material to allow for the identification of key themes and interpretations related to the integration of SW into SAPV-teams, without imposing predetermined assumptions on the data.

The interview guide was developed to balance structure with flexibility. It was designed with open-ended, narrative prompts and key phrases to provide order while still allowing for the interviewees’ natural storytelling.^17^ The guide, by J.W., contained 14 questions organised into thematic blocks, covering topics such as the participants’ professional backgrounds, daily work routines, the impact of SW in specific cases and future perspectives on SW in palliative care. The iterative development of the guide involved a process of collecting, verifying, sorting and refining questions,^17^ ensuring a logical transition from general to specific issues. The first interview was supposed to be a pre-test, but there were no adjustments needed, so we chose to utilise the complete interview for the analysis.

Follow-up questions were employed to explore specific topics in greater depth, fostering a balance between flexibility and comprehensiveness in the study.^12^ All interviews were conducted in person by J.W. during regular work hours in the offices at the hospital, with no participants declining to take part. No interviews had to be repeated. Audio recordings were transcribed verbatim via Trint transcription software. The transcripts were not returned to the interviewees for review or revision. Interviews averaged 30–45 min in duration, with no need for repetition. Field notes were taken by the interviewer during the sessions. Saturation was evaluated by the research team, leading to the planned conclusion of data collection after eight interviews. To maintain data security, all files were saved on an external drive accessible only to the J.W., the solo data coder.

### Data analysis

Sequence analysis was used to interpret the meaning of the interviews. The results are interpreted based on the inductively selected sequences and brought into context with the other interviews. According to Reichertz,^18^ the calibration must be set before a sequence analysis, that is, how small or large the sequences to be examined should be. We chose the third ideal-typical calibration of the sequence analysis that increases the range of meaning units towards the meaning and action units set by the speakers because we wanted to interpret social phenomena as precisely as possible. This approach originates from the ethnology method of Clifford Geertz’s thick description, which attempts to condense individual observations into general statements. In doing so, the re-construction of what the interviewees construct on-site^15^ is essential. Geertz’s concept of culture is understood as this web of meaning rather than laws. Thus, ethnological observations are always also interpretations, not pure descriptions, but ‘thick description’. It combines three levels of abstraction: empirical (people’s actions or utterances), meaning (the context of these activities) and metanarrative (unconscious frameworks of action). Geertz argues that cultural meanings differ and that researchers must make a specific intellectual effort to understand different meanings.^19^

Sequence analysis was developed in 1979 by Ulrich Oevermann and has evolved since then. In this paper, it is used as ‘applied hermeneutics’^18,20^ an attempt to reconstruct the meaning of human actions, including speaking and writing sequentially.

Analysing the interviews sequentially means starting with a detailed interpretation of a sequence from the first interview that leads to the establishment of an interpretive hypothesis, which is then tested by case-immanent and cross-comparison with the other interviews. The interview focus was on how SW is present in the SAPV-team and what special tasks it takes on in the interprofessional team.

## Results – Potentials and challenges of SW in SAPV

This research is based on non-standardised, qualitative interviews with four nurses and four physicians from the SAPV-team at Heidelberg University Hospital. The interviews were conducted and analysed by the same researcher, and participants did not provide feedback on the findings. The results are organised into five distinct sections to offer a clear overview of the various insights, with two tables included at the end to summarise key points.

### Experiences and responsibilities of social workers

For some interviewees the SW is seen as a useful support, but one that has a lower status compared to the medical staff. They perceive the social worker as an on-call specialist who is primarily responsible for the socio-medical needs of patients and relatives but is not seen as a core member of the medical team. This attitude becomes clear from first, the attribution of solely and exclusively socio-medical issues to the responsibilities of the social worker, and second, the perception of the power to delegate a task assumed as simple but time-consuming instead of the necessity of entrusting a specialist who is a colleague. The following quote from interviewee 1 served as the starting point for all subsequent interviews utilising the sequence analysis approach:

SW takes on a very important role here, because we can delegate many tasks that fall under the socio-medical field and these are actually very many socio-medical matters that are a burden for patients and their relatives. It is even more relieving for us when we can also offer the supportive assistance of our social worker on site. (I1, 8)

Further, what is implied is that the SW does not necessarily have to be directly on-site, because medical and nursing care is sufficient to cover the most important part of care, and the term cooperation was not mentioned at all. However, because the concerns are dealt with very quickly and then the patients quickly realise that something is happening or relatives (I1, 8) indicate patient satisfaction when they realise that something tangible is happening in the care process and that they can rely on all members of the SAPV-team. SW appears to take on a coordinating role in helping patients and relatives with many organisational needs as well as financial and socio-legal issues, which is appreciated also by the rest of the SAPV-team.

This analysis, derived from interviews with various healthcare professionals, delves into the experiences and responsibilities of social workers within the palliative care setting, highlighting their indispensability and the nuances of their involvement. One interviewee distinguished between SW in palliative care and neurology. Social workers in palliative care are more engaged and involved in the daily operations compared to their neurology counterparts with significant exchange leading to optimal patient solutions. That being said, when asked about the actual tasks of the social worker, only advice on social law issues or arranging care services is mentioned (I1, 24):

Overall, contact with SW was much more sporadic and less frequent in neurology. Whereas here in our palliative care team, there is a lot of lively exchange, and you can be sure that the best possible solution for the patient is attempted. (I1, 22)

The gap between understanding SW as part of the team and the tasks associated with it, is obvious in some team members who are aware of the importance of SW but have difficulty with its integration into the concept of interprofessional care.

### Perceived SW tasks and integration challenges

As soon as the social worker is missing from the SAPV-team there are serious gaps in care, which the medical staff particularly emphasise (I2, 43). One nurse described SW as indispensable support: ‘This support is comparable to an important piece of the puzzle, without that piece, the puzzle can’t be finished’ (I6, 66). SW is perceived as crucial in a variety of situations, often just for advice or exchange.

Two main tasks of SW are identified: handling matters with authorities and coordinating within the SAPV-team. The social worker acts as a ‘networker, providing contacts to patients as soon as they [patients and families] contact us’ (I3, 16). The urgent need for support by SW typically occurs during home visits performed by nurses and physicians when patients and relatives are overwhelmed with paperwork. The specialist nurses determine the required support on-site and refer this information to the social worker. An important contribution to this function is that the social worker is present every day, although part-time, and so has the most complete picture (I5, 36) of the specific patient, which distinguishes them from other professionals in the team.

One interviewee emphasises the need to integrate this expertise more robustly into daily work rather than merely delegating tasks, for example, ‘if the social worker’s position were to be eliminated, a core competence of specialist palliative care would be lost’ (I2, 43). The social worker’s role as a coordinator within the team ensures patients are referred to appropriate services and that their needs are addressed promptly. One appreciates SW as ‘an extremely great help’ (I2, 14), especially in navigating bureaucratic processes and providing support to patients, relatives and SAPV colleagues. Delegating chores to social workers that a nurse or doctor could accomplish themselves but are perceived as unpleasant or routine, leads to decreased esteem for social workers.

Three interviewees advocate for assistance in all parts of the Total Pain concept, emphasising that specific patients’ capacity to maintain what they perceive as ‘their normal lives’ is critical (I3), regardless of the teams’ or outsiders’ norms. Further, SW facilitates discussions about financial issues directly with the patients, suggesting a more approachable interaction (I5, 38). Highlighting that patients’ needs in SAPV are not medical only (I6, 22).

One interviewee also acknowledges the considerable benefit that collaboration within the team between medical staff and SW brings to patients (I5, 16). The interviewee also states that social workers in hospitals were often guided by the nursing staff or by good physicians who are aware of the patient’s perspective (I5, 26). This statement acknowledges that not all healthcare professionals possess the skills required to organise the tasks to do for a patient. Actually, one physician wished for a reference book to clearly define SW responsibilities, particularly in non-medical areas, to better inform the team (I1, 36).

Nonetheless, the interviews revealed that certain members of the SAPV-team find social workers necessary, but do not incorporate them into the team. This could be due to the fact that they actively experience power imbalance. Those interviewees mentioned that social workers were responsible for advice on social law issues, but also for arranging nursing services, and other time-consuming tasks, thus, viewing SWs essentially as assistants (I8, 18). In contrast, one nurse wondered if contacting nursing services should not be a SW responsibility, implying that patients and their informal healthcare givers could handle it themselves (I6, 62). These insights underscore the critical role of interprofessional work in supporting patients with life-ending illnesses.

### Addressing psychosocial needs

SW is important to one of the physicians because often patients prioritise psychosocial issues over medical ones, for example, wanting to stay at home and at the same time not being a burden to their relatives (I4, 18). According to the interviewee, only 50% of SAPV tasks are medical, with the other half being psychosocial, often only requiring medical staff to step in at the final stages (ibid, 20). One nurse considers SW essential and relieving, directly benefiting patients. Another nurse observed that having several professional groups also helps patients choose who they want to share difficult or embarrassing issues:

But I really appreciate that you exist and also the way of writing with a task, or, or and because I also find it very relieving, and it and I just see what a benefit it brings out there for the patients. (I5, 16)

The tasks of a social worker and a psychologist were also compared twice. Interviewee 2 believes that the SW can cover more than a psychologist, ‘because it is clear that the area of application is simply larger and also more practical’ (I2, 39). Interviewee 4 on the other hand, believes the two professions are more comparable given that ‘in my experience [social workers] are also good psychologists’ (I4, 10). According to the two physicians, the professional groups in palliative care merge, with SW capable of working in a variety of fields, including psychology.

### Organisational and hierarchical challenges

The members of the SAPV-team can commission and track orders with the support of an electronic patient chart. Also, in this area the social worker’s presence and efficiency were appreciated, noting that tasks are completed quickly, relieving the team (I5, 24) and mentioning that they can contact the social worker for urgent concerns, benefiting from the team’s proximity and ease of communication (I8, 12).

Some interviewees addressed the need to extend SW hours until the afternoon and ensure reliable sickness and vacation coverage to maintain swift task completion and direct communication between healthcare professionals and social workers. Some physicians and nurses also desire the integration of additional professional groups, such as psychotherapists, physiotherapists and counsellors, to supplement SW and provide more comprehensive team support.

### SAPV without SW

A central task of SW in SAPV is the support of relatives, in particular where physicians and nurses are at limits with their specialities. The specific knowledge and skills of social workers ensure that the needs of relatives are sufficiently considered:

The assistance of relatives in this regard is absent because we frequently reach our limitations with our specialist expertise. There are some things that cannot be clarified from a medical or nursing perspective. (I1, 18)

In addition, the tasks to do quickly add up if there are no social workers available. Many administrative tasks are urgent, such as registrations in nursing homes and applications for legal support and cannot simply be put off until the following week. The absence of SW means that important tasks are left undone, this then means that patients’ care does not run smoothly.

Most interviewees experience a clear beneficial effect of SW also on symptom control. Without the social worker, it would be ‘more difficult to control the symptoms. All our patients would certainly be in more pain’ (I3, 46). The crucial need for SW in palliative care is underlined by one physician, stating that a patient receiving SAPV could have perished without it: ‘I suppose I can think of a patient who were sure, but it’s also evident that SW saves lives’ (I4, 12). Effective symptom control is insufficient if the patient’s household or social condition is not rectified. Medical symptoms can be controlled with pharmaceutical medicine, but patients require social medicine to address problems such as the absence of relatives (I2, 43).

Another important role of SW is to relieve the medical staff. Social workers discuss with relatives and deal with socio-legal aspects that cannot be fully taken on by other co-workers. The workload increases, and patient care is impaired without the support of social workers. Nurses and physicians emphasise that they do not have the expertise and experience to fully take on the tasks of SW.

According to one interviewee, without social workers, the quality of care and follow-up would suffer dramatically, with physicians and nurses taking on SW responsibilities. The involvement of relatives would be more difficult while also increasing the workload. They emphasise the importance of SW, which is more critical for palliative patients than in other wards:

Is already more necessary for palliative patients [. . .] than it is on other wards, [. . .] and that’s why I believe it’s an important job description in our profession; I’m not saying this because you’re a social worker. (I7, 78)

In this context, interviewees found interdisciplinary cooperation to be essential to deal with uncertainties in patient care: ‘I have a feeling I’m not alone. I can always reassure myself with the physician and social worker because there are so many variables’ (I6, 18). Without this support, they would consider resigning (ibid., 67). This threat of resignation illustrates the critical role of SW to balance the workload of SAPV-teams with expertise, as colleagues cannot compensate for the specialist tasks of SW (I6, 71).

The following four sections provide the research’s key findings which are based on the interview guide; please see Tables 2 and 3 for a brief overview.

**Table 2.** Challenges of SW in SAPV.

Topic	Results
Experiences and responsibilities of social workers	- Lower status than medical staff - On-call specialist - Medical staff only knows the basics about SW daily tasks
Perceived SW tasks and integration challenges	- Symptom control goes beyond medical therapies - Missing knowledge about SW tasks - Incorporation into the team SW is seen as an assistant
Addressing psychosocial needs	- Overlapping responsibilities in palliative care
Organisational and hierarchical challenges	- Working hours are too short - Seeking other professional groups beyond SW
Palliative care without SW	- Urgent tasks quickly add up - Need for interdisciplinary cooperation medical staff would resign without a social worker in the team

SAPV, specialist palliative home service; SW, social work.

**Table 3.** Potentials of SW in SAPV.

Topic	Results
Experiences and responsibilities of social workers	- Takes on coordinating role with organisational needs - More engaged and involved in the daily operations
Perceived SW tasks and integration challenges	- Indispensable support - Crucial in a variety of situations - Often just for advice and/or exchange coordination within the team and authorities
Addressing psychosocial needs	- Patients prioritise psychosocial issues over medical ones - Directly benefiting patients, several professional groups help patients choose who they want to share difficult or embarrassing issues with
Organisational and hierarchical challenges	- Tasks are completed efficiently, benefiting from the team’s relationship and ease of contact
Palliative care without SW	- Positive on symptom control (concept of total pain) to relieve medical staff

SAPV, specialist palliative home service; SW, social work.

## Discussion

We explored the attitudes towards SW of nurses and physicians providing specialist palliative home care in Germany. The study identifies multiple reasons for integrating SW more thoroughly into SAPV-teams, including the expectations of medical personnel, and the perceived needs of patients and relatives. The results highlight the indispensable role of social workers in enhancing the quality of patient care and reducing the workload of medical staff. Nevertheless, from the perspective of the SW profession, there is a clear need for better integration and recognition of their discipline within SAPV-teams to optimise interprofessional collaboration and patient outcomes. Our study clearly shows that there is still a tension between the attributions of the health professions to SW and the actual tasks of SW in an interprofessional team.^4^ Physicians and nurses in our study often regard social workers as supportive, especially for non-medical tasks and discussions with relatives, but desire a clearer profile and greater integration of SW within the process of care. That said, some interviewees had little, or no notion of what SW entailed, and how they could incorporate it into their daily workflows. Conversely, the other interviewees articulated unmet needs related to SW. On the one hand, the SW’s working hours are deemed too short, and more presence of the social worker is required during home visits to patients according to the medical staff; on the other hand, the medical staff is unsure about the social workers’ competencies and responsibilities. Yet, social worker Sindy Herrmann has already published an open-access list of the key competencies of SW in this special field of activity on the website of the German Association for Palliative Medicine (DGP), which includes 23 such skills and could certainly be considered a reference book. The fact that this easily accessible information was not retrieved may point to a lack of time or motivation, illustrating the distance of actual integration of SW into SAPV.^21^ Interestingly, it does not appear to be an entirely new challenge for social workers around the world, which is why the International Federation of Social Workers established a revised definition of SW in 2014. According to this, SW promotes social transformation and growth, as well as people’s autonomy and self-determination, in accordance with social justice and human rights principles.^22^ The expertise of counselling and assistance choices becomes tremendously beneficial. In the face of poverty, disadvantage, exclusion and excessive demands, social problems and problem solutions are important.

Nevertheless, social workers are deemed essential, providing expertise in handling psychosocial and socio-legal issues. However, the findings of our study indicate that the integration of SW into SAPV is still in its early stages. According to Student^23^ SW, particularly outreach SW, is a highly useful part of the palliative care approach and teamwork since it can operate across the entire system. According to several studies,^24–26^ the hospital structure has a strong hierarchy based on professional groupings, as well as a poorly formed culture of cooperation. Aside from the legal classification of employees into physicians, nursing and administrative staff based on occupational groups, there are further informal gradations within these professional groups as well as the power of physicians to give medical instructions to nursing staff. The issue is therefore not only to integrate social workers into regular clinical practice but also to encourage nursing staff and physicians to collaborate in an interprofessional manner. Here, the position of SW operating across the entire hospital system and across healthcare sections could be a facilitator.

Palliative care, as defined by the WHO, ‘involves a range of services delivered by a range of professionals that all have equally important roles to play – including physicians, nursing, support workers, paramedics, pharmacists, physiotherapists and volunteers – in support of the patient and their family.’^2^ Even though the WHO definition as well as the studies date back more than two decades, our sequence analysis yielded similar results. The medical staff regards social workers as knowledgeable about social medicine and valuable assistants, sometimes even on an equal footing with physicians. Yet, social workers tend to be seen as on-call specialists for certain duties rather than core team members. According to Kiepke-Ziemes and Wasner,^4^ social medicine focuses on the social determinants of health whereas SW prioritises comprehensive treatment for individuals. Social workers in palliative care serve as points of contact, contribute social perspectives and moderate family conferences. They can benefit the institution through administrative tasks and public relations. Despite its increasing integration in hospitals, the role of SW in palliative care processes in Germany is still unclear and insufficiently regulated by law, according to Pankofer.^4^ A curriculum developed by Schütte-Bäumner and Lehmann^27^ for professionals in hospice and palliative care emphasises the need to specify the perspective of SW in trans- and interprofessional contexts.

An important finding is that medical knowledge alone is insufficient to meet the needs of palliative care patients. Social workers are necessary to reduce the burden on patients and relatives, contributing significantly to pain relief and overall care quality. The integration of SW into SAPV-teams is hindered by the allocation of medical staff, the need for more extensive training and the presence of social workers. A change in the law to establish psychosocial professional groups alongside physicians and nurses in SAPV-teams is recommended to address these challenges and meet the growing care demands. Is SW more comparable to the roles of physicians and psychologists, as this could help medical staff to understand its purpose? Or does SW only function effectively when guided and instructed by good physicians and nurses as one interviewee suggested? One respondent, for instance, would counter that the individual SAPV-team should function cohesively and occasionally assume the responsibilities of colleagues, negating the need for a large reference book. However, each member of the team should be aware of the unique skills held by each member of the professional group, as according to Neupert,^28^ the specific structures of palliative care in particular are distinguished by a high degree of differentiation based on the needs of the addressees.

The responsibilities of medical personnel are no barrier to the integration of SW into SAPV. That said, we believe that more time and thorough training are required for medical experts to become acquainted with the other professional groups, as well as additional staff, which would provide more opportunities to alleviate patients, families and team members. In our opinion, a greater presence of social workers may illustrate the required time and expertise devoted to individual patients’ difficulties, which should lead to better goal achievement, that is, quality of life until the end of life.^29^ As a result, the reduction of the burden on patients and relatives is ultimately what all respondents in the study’s interviews desired and, to some extent, expected from SW.

An important step in this approach would be to change the legal regulation to allow for the adaptation of the SAPV framework agreement (Section 132d (1) sentence 1 of SGB V). Only recently, at the Intergroup for Hospice in the German Bundestag on April 23, 2024, DGP Vice President Andreas Müller argued for the establishment of psychosocial professionals as a third professional group alongside physicians and nurses to manage the enormous difficulties in psychosocial care in SAPV.^30^ This demand needs urgent action because the number of people in need of care has doubled in the last 30 years.^6^ More people in need of care also means more people who would benefit from SAPV, because those people at the end of life with increased care needs should get access to appropriate interprofessional care.

## Strengths and limitations

Unfortunately, there is little literature on the subject of SW and palliative care in Germany. As a result, we felt this study was even more relevant and urgent to scientifically prove that this profession has a basis and is necessary for palliative care. This is the first study exploring the attitudes towards SW of SAPV nurses and physicians. An important limitation of this study is the potential social desirability bias and issues regarding reflexivity due to the interviewer being a member of the SAPV-team. We addressed this risk with participatory observation by two external researchers experienced with the technique.

It was not the aim of the study to make simple generalisations from a case study about the status of SW integration in SAPV. However, we could show that in this field, the professional demands and self-perception of SW are in tension with the medical professional demands on palliative care. The established medical perspective still dominates here. In this respect, the integration of SW – if it is not to remain a vicarious agent – depends on the changing of traditional external attributions as long as there is no other regulatory framework.

## Conclusion

SW is a necessary component of SAPV, yet it requires additional resources and training for medical personnel. Furthermore, there is insufficient funding for this third professional group to provide seamless treatment and keep professionals in this field from being displaced by laypeople.

We found out that medical staff members are in charge of determining if and how much SW is included in routine care. Physicians and nurses provided recommendations regarding extending the working hours of the social workers, requiring sickness and vacation coverage. Furthermore, they wished to integrate other professionals, such as psychotherapists and counsellors, to provide comprehensive support and have a social worker accompany the team on home visits.

Social workers need to clearly define their tasks and responsibilities. Medical staff rely on social workers for patient-oriented SAPV, highlighting the need for their integration into the team. This study, though not representative of all SAPV-teams in Germany, points to the need for larger sample sizes in future research. The aim would be to find out why the process of integration of SW in SAPV is progressing so slowly. Interviews with social workers and service managers across Germany may provide deeper insights into the challenges and arguments for social service integration. In addition, interviews with managers could clarify arguments and challenges regarding the decision to establish a social worker in their team.

## Declarations

**Ethics approval and consent to participate:** Not applicable, because due to the regulations of Heidelberg University the current studies do not need formal ethics approval and no patients were interviewed in this study. The consent of the participants in a written way also ahead of the interviews.

**Consent for publication:** Not applicable, because all the data is anonymous.

**Third party submissions:** Not applicable because does not exist.

**Writing assistance:** Not applicable because does not exist.

**Availability of data and materials:** The transcripts of the interviews will also be sent to the submitting website; we simply need to note that both the interviews and the transcripts were conducted in German.
